# Assessment of Risk Factors for Coronary Artery Restenosis and Patient Survival During the COVID-19 Pandemic

**DOI:** 10.3390/healthcare13101175

**Published:** 2025-05-18

**Authors:** Lyudmila Pivina, Andrey Orekhov, Gulnara Batenova, Diana Ygiyeva, Tatyana Belikhina, Maksim Pivin, Altay Dyussupov

**Affiliations:** 1 Department of Emergency Medicine, Semey Medical University, Semey 071400, Kazakhstan; lyudmila.pivina@smu.edu.kz (L.P.); gulnara.batenova@smu.edu.kz (G.B.); diana.ygiyeva@smu.edu.kz (D.Y.); 2 Department of Internal Medicine, Semey Medical University, Semey 071400, Kazakhstan; 3 Board for Strategic Development, Scientific and Educational Activities, National Research Oncology Center, Astana 020000, Kazakhstan; 4 Medical Center Hospital of the President’s Affairs Administration of the Republic of Kazakhstan, Astana 020017, Kazakhstan; pivin97@mail.ru; 5 Board, Semey Medical University, Semey 071400, Kazakhstan; altay.dyusupov@smu.edu.kz

**Keywords:** in-stent restenosis, stenting, coronary artery disease, COVID-19, risk factors, survival

## Abstract

**Background**: In-stent restenosis of coronary arteries is a significant problem in interventional cardiology. Inflammatory processes in the arterial intima play a key role among the well-known risk factors for restenosis. The COVID-19 pandemic has contributed to the development of inflammation and the activation of the coagulation system. The aim of this study was to assess the risk factors for coronary artery restenosis and patient survival during the COVID-19 pandemic. **Materials and Methods**: We performed a cross-sectional study on a targeted sample of patients with coronary artery disease who underwent repeat myocardial revascularization (931 patients). The main study group, consisting of patients with coronary artery stent restenosis, included 420 patients (38.5% had previous COVID-19). The control group included 511 patients without stent restenosis (20.9% had COVID-19). **Results**: The results of multiple logistic regression analysis showed that the odds ratio (OR) for COVID-19 was 2.29 (95% CI 2.78–3.19) (*p* < 0.001), and the OR for C-reactive protein (CRP) was 1.08 (95% CI 1.002–1.013). The average hospital survival time for subjects with prior COVID-19 (N = 269) was 9.53 ± 0.106 days (95% CI 9.32–9.74), while for those without COVID-19 (N = 662), it was 9.89 ± 0.032 days (95% CI 9.83–9.96) (*p* < 0.001). The one-year survival time was 316.7 ± 6.982 days (95% CI 303.0–330.4) for the COVID-19 group and 340.14 ± 3.243 days (95% CI 333.8–346.5) for the non-COVID-19 group (*p* < 0.001). **Conclusions**: The main risk factors for in-stent restenosis were COVID-19 and elevated CRP levels. The average survival time in the group with prior COVID-19 was statistically significantly lower than in patients without COVID-19, both during the hospital stay and within one year after repeated revascularization.

## 1. Introduction

Coronary artery restenosis is characterized by a narrowing of the arteries by 50% or more after previous myocardial revascularization (angioplasty or stent placement). According to various studies, the main risk factors for restenosis include nicotine addiction, which promotes inflammation and damage to the vessel walls [1,2]; diabetes mellitus [3]; hyperlipidemia with the formation of cholesterol plaques in the vessel walls; arterial hypertension; male gender; concomitant infectious and inflammatory diseases [2]; errors in the use of antiplatelet therapy [4]; the quality and type of stent; and the type of drug coating on the stent [5]. These risk factors can act both separately and in combination, increasing the likelihood of coronary artery restenosis after intervention [6].

Coronavirus infection, caused by the SARS-CoV-2 virus, can significantly affect hemostasis and lead to hypercoagulation, increasing the risk of thrombosis and myocardial infarction. The mechanisms by which coronavirus causes hypercoagulation and thrombosis include several factors. These include direct damage to the coronary vessel wall through viral infection, inflammation, and activation of the coagulation system due to the release of large amounts of proinflammatory cytokines, which leads to an increase in fibrinogen levels and thrombus formation [7,8]. Systemic inflammation during viral infection contributes to increased vascular permeability, creating a prothrombotic environment. Additionally, tissue hypoxia, characteristic of COVID-19, can activate genetically determined mechanisms of thrombosis [9]. This infection is also characterized by the suppression of fibrinolysis processes and the direct activation of platelets, especially in small vessels, which disrupts microcirculation in vital organs, leading to multiple organ dysfunction and acute respiratory distress syndrome [10].

The survival of patients with myocardial infarction due to coronary artery restenosis after coronavirus infection depends on many factors, including the patient’s age, the presence of comorbidities, the severity of both the infarction and the coronavirus infection, as well as the quality of medical care provided. Patients who have recovered from COVID-19 have an increased incidence of complications such as acute heart failure and arrhythmias, and a higher risk of cardiogenic shock. This group of patients also has an increased risk of thromboembolic complications, such as stroke or reinfarction, which reduces the chances of successful recovery and may decrease survival [11,12]. Studies show that patients with myocardial infarction after COVID-19 have a higher risk of death in the short and medium term [13]. In general, these patients require more careful medical supervision, intensive care, and ongoing monitoring of the heart and blood vessels. The aim of our study was to assess risk factors for coronary artery restenosis and patient survival during the COVID-19 pandemic.

## 2. Materials and Methods

### 2.1. Characteristics of the Study Groups

#### 2.1.1. Study Design

The study design was a cross-sectional study conducted on a target sample of patients with coronary artery disease who underwent repeat myocardial revascularization at two hospitals (Semey Emergency Hospital and Semey Medical University Hospital) from May 2020 to May 2023. A total of 931 patients were included in the sample. The main study group, which had coronary artery stent restenosis, consisted of 420 patients, of which 162 (38.5%) had a history of coronavirus infection. The control group included 511 patients who underwent repeat myocardial revascularization without stent restenosis; of these, 107 (20.9%) had a history of coronavirus infection. Exclusion criteria: patients with autoimmune systemic or acute inflammatory diseases, psychiatric disorders, cancer patients, and patients who refused to participate in the study.

After risk stratification, all patients underwent coronary angiography (CAG), followed by myocardial revascularization with stenting. A study participant card was created for each patient. All participants were informed about their inclusion in the study, and they were made aware that the results would be published in a scientific journal while ensuring the confidentiality of their information. Written consent was obtained from each patient to participate in the study.

Vital status and adverse outcomes (death, development of myocardial infarction, coronary artery stent restenosis) were monitored for one year using telephone interviews with the subsequent confirmation of information using electronic information systems.

#### 2.1.2. Outcomes

As a primary outcome, we considered the presence of coronary artery restenosis, as well as hospital survival and survival within a year in patients with repeat myocardial revascularization, depending on the presence of restenosis and a history of coronavirus infection. Secondary outcomes included the clinical and laboratory parameters associated with coronary artery restenosis.

#### 2.1.3. Age Distribution

The age distribution of all patients included in the study was normal. The average age of all participants was 64.3 ± 8.2 years. For women, this figure was 67.1 ± 10.5 years, while for men, the average age was 63.4 ± 9.9 years.

#### 2.1.4. Collection of Clinical and Laboratory Parameters

Clinical data were collected from an electronic medical database, including demographics, clinical data, comorbidities, imaging results, laboratory tests, clinical outcomes, previous myocardial revascularization, and history of coronavirus infection. All diagnoses were made by experienced specialists. All recorded events were reviewed from hospital electronic records and assessed by two cardiologists through consensus. Venous blood samples were collected from all patients within 10 min of admission. Laboratory tests included complete blood count, high-sensitivity troponin I, D-dimer, creatine kinase (CK), creatine kinase-MB (CK-MB), serum creatinine and glucose, ESR, C-reactive protein (CRP), alanine aminotransferase (ALT), aspartate aminotransferase (AST), and fibrinogen. Evidence of previous coronavirus infection was obtained from patient history and laboratory parameters, including IgG and IgM antibodies against the SARS-CoV-2 virus and the detection of COVID-19 RNA using polymerase chain reaction (PCR). Current coronavirus infection was confirmed by PCR analysis and IgM titer, and previous infection by IgG titer. There were no cases of COVID-19 death among the patients included in our study. All cases of coronavirus infection in the study groups were comparable in severity and were categorized as mild and moderate.

Of the 931 patients who underwent PCR testing, only 19 patients tested positive, of which 10 patients had coronary artery restenosis.

### 2.2. Statistical Analysis

Descriptive statistics were used in this study. For all continuous variables, the mean and corresponding confidence intervals were calculated depending on the type of data distribution. For variables with non-normal distributions, the median and interquartile range were determined. Qualitative variables were analyzed by calculating absolute and relative frequencies. For categorical variables, data were presented as absolute and relative numbers. The significance of differences between groups for categorical data were determined using the Chi-square test (χ^2^). For quantitative data, central tendencies were measured. The comparison of laboratory parameters between patient groups was performed using the nonparametric Mann–Whitney U test for samples with an asymmetric distribution. Nominal variables were compared using the Pearson χ^2^ test, and ordinal variables were analyzed using the Kendall Tau test.

Paired linear regression analysis was used to assess the correlation between parameters. The relationship between risk factors was studied using multiple linear regression analysis. Odds ratios were calculated to assess the contribution of each risk factor to the development of restenosis. The survival function of patients was assessed using the Kaplan–Meier method, with the survival function displayed as a descending step line; the values between observation points were considered constant. The survival analysis was performed using Cox regression, which predicted the risk of an event in the studied sample and evaluated the effect of predetermined independent variables (predictors) on this risk. Statistical significance was set at *p* < 0.05. Statistical calculations were performed using SPSS version 20.0 software (IBM Ireland Product Distribution Limited, Dublin, Ireland).

## 3. Results

Demographic characteristics such as gender and age did not show statistically significant differences between the study groups. Among the comorbidities, only coronavirus infection was statistically significantly more common in the main group of patients than in the control group (*p* < 0.001). The median left ventricular ejection fraction (LVEF), as assessed by echocardiography, was 52% in both study groups. Regarding laboratory parameters, statistically significant differences between the groups were found only for the level of C-reactive protein (*p* < 0.003) (Table 1). According to anamnesis and the levels of IgG and IgM antibodies to coronavirus, all patients with a history of COVID-19 had contracted the virus before being admitted to the hospital for coronary artery disease.

Table 2 presents the odds ratios for factors associated with coronary in-stent restenosis in individuals who underwent repeat myocardial revascularization (N = 931), as calculated using binary logistic regression. Analysis of the data from univariate regression indicated that the odds ratio for coronary artery restenosis in patients with diabetes mellitus was 1.137, although this result was not statistically significant. Age, gender, and left ventricular ejection fraction did not significantly affect the development of stent restenosis. Regarding laboratory parameters, a statistically significant increase in the odds of coronary in-stent restenosis was observed only for C-reactive protein (*p* = 0.002). The greatest increase in the odds of coronary artery restenosis was seen in patients with a history of coronavirus infection, with an odds ratio of 2.378 (*p* < 0.001) (Table 2).

The results of multiple logistic regression analysis showed that in patients with previous COVID-19, the odds of developing restenosis increased by 2.29 times. With an increase in serum C-reactive protein, the odds of developing restenosis increased by 1.17 times (Table 3).

Comparison of risk factors for adverse cardiovascular events in groups of individuals depending on the presence of COVID-19 in their medical history demonstrated a statistically significantly more frequent presence of coronary artery restenosis in the group of patients who had COVID-19, as well as statistically significant differences in laboratory parameters such as the platelet–neutrophil ratio, APTT, fibrinogen, D-dimer, AST, CPK and MB CPK, glucose, and CRP in the study groups (Table 4).

**Figure 1:**
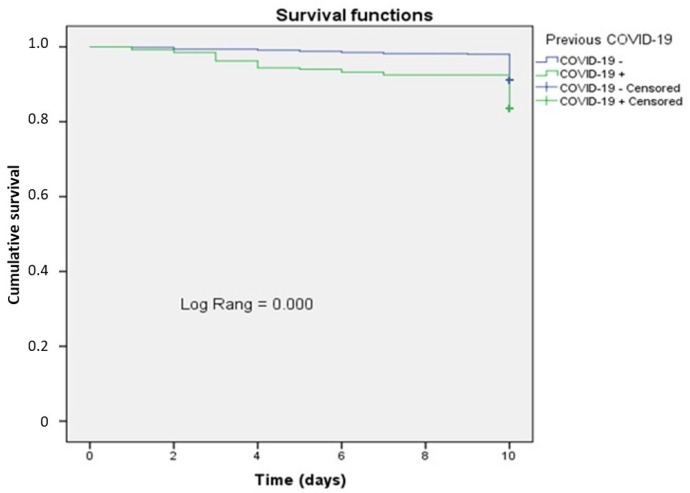
Overall hospital survival curve depending on the previous incidence of COVID-19.

During the entire study period, 103 patients died, of which 54 (12.9%) were in the main group with coronary artery restenosis and 49 patients (9.6%) were in the control group (*p* = 0.117). The cause of death in all cases in both groups was myocardial infarction. Considering that coronary artery restenosis is directly associated with adverse outcomes of myocardial infarction, we further analyzed overall hospital survival in the subjects (N = 931) based on the presence of COVID-19 using the Kaplan–Meier method. The average survival time in subjects with a prior history of COVID-19 (N = 269) was 9.53 ± 0.106 days (95% CI: 9.32–9.74), while in the group without COVID-19 (N = 662), it was 9.89 ± 0.032 days (95% CI: 9.83–9.96). The median survival time in both groups was not reached. The differences in overall survival during the hospital stay were statistically significant (χ^2^ = 12.144; *p* < 0.001) (Figure 1). In the group with COVID-19, 20 fatal outcomes were recorded (7.5%), while in the group without COVID-19, there were 13 such cases (2.0%) (*p* < 0.001). When assessing the risk of a fatal outcome based on the presence of COVID-19 during the hospital stay, we found a fourfold increase in the risk of an unfavorable outcome in the group of patients with a history of COVID-19. These results indicated a higher risk of fatality in patients with repeat myocardial revascularization following coronavirus infection and a lower survival rate for this group in the near future.

**Figure 2:**
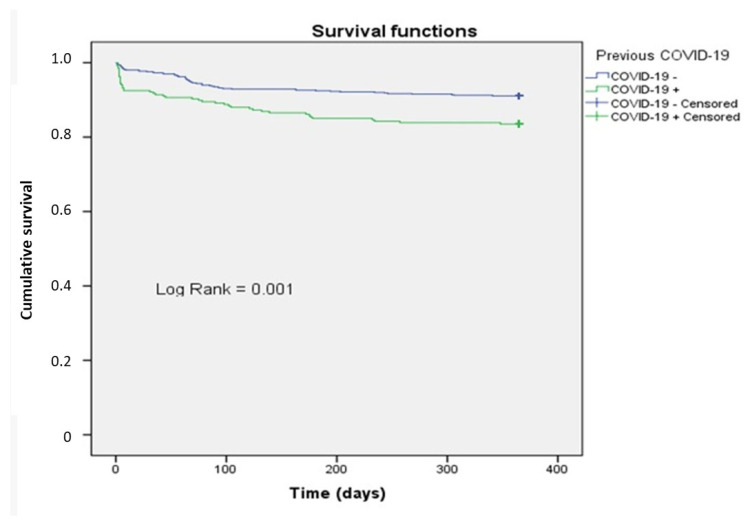
One-year survival curve based on previous incidence of COVID-19.

When assessing the survival of the study patients (N = 931) over the course of one year depending on the history of COVID-19 and using the Kaplan–Meier method, the average survival time in the group with a history of COVID-19 (N = 269) was 316.7 ± 6.9 days (95% CI: 303.0–330.4), while in the group without COVID-19 (N = 662), it was 340.14 ± 3.2 days (95% CI: 333.8–346.5). The differences between the groups were statistically significant (χ^2^ = 11.611; *p* < 0.001) (Figure 2). In the group of patients with a history of COVID-19, 44 cases of adverse cardiovascular events (16.5%) were recorded, compared to 59 cases (8.9%) in patients without COVID-19 (*p* < 0.001). The evaluation of the risk of developing an adverse outcome over the year showed a twofold increase in the risk of death in the group of patients with a history of COVID-19.

**Figure 3:**
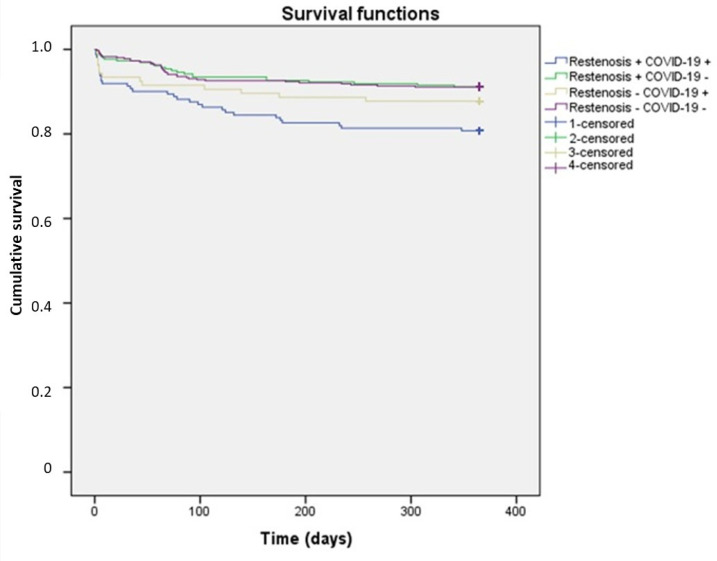
Characteristics of the one-year survival function of patients depending on the presence of coronary artery restenosis and COVID-19.

The characteristics of one-year survival in patients, depending on the presence of coronary artery restenosis and COVID-19, are presented in Figure 3. The patients are divided into four groups: (1) those with coronary artery restenosis and a history of COVID-19; (2) patients with restenosis without COVID-19; (3) patients with repeat coronary artery revascularization without restenosis, but with COVID-19; and (4) patients with repeat coronary artery revascularization without restenosis and without COVID-19. In the first group, the average survival time was the shortest: 309.5 ± 9.4 days (95% CI: 290.9–328.01). In the third group, this value was 327.7 ± 10.1 days (95% CI: 307.9–347.4). In the second and fourth groups, where patients did not have a history of COVID-19, the average survival times were the highest and were nearly identical: 340.9 ± 5.1 days (95% CI: 330.9–350.9) and 339.6 ± 4.2 days (95% CI: 331.4–347.9), respectively.

When assessing the relationship between overall survival and the risk factors studied using the Cox regression method, the following proportional hazards model was obtained:

h_i_(t) = h_0_(t) × exp(0.701 × XStatus_COVID-19: Presence of COVID-19 − 0.028 × XLVEF + 0.027 × XAge)

h_i_(t)—predicted risk of adverse cardiovascular events, XStatus_COVID-19: Presence of COVID-19.

The adjusted hazard ratio (HR) for the factor of having a history of COVID-19 was the highest—2.017 (*p* < 0.001), and with increasing age, the risks increased by 1.028 times (*p* = 0.006) (Table 5). The relationship between the ejection fraction and the risk of an unfavorable outcome was negative—with an increase in LVEF by 1%, the risks decreased by 1.029 times; HR = 0.972 (*p* = 0.004).

Thus, the main prognostic factors for the development of adverse cardiovascular events within a year after myocardial revascularization in our study were previous coronavirus infection, the patient’s age, and a decrease in the left ventricular ejection fraction.

## 4. Discussion

The results of our study demonstrated that the main statistically significant risk factors for coronary artery restenosis were previous COVID-19 infection (OR 2.378; 95% CI [2.778; 3.191]) and C-reactive protein (OR 1.009; 95% CI [1.0003; 1.015]). A review of the literature was conducted to compare the risk factors for restenosis in our study with those found in similar studies. In a study by Chinese researchers involving 141 patients with unstable angina, stent restenosis ≥ 50% was observed in 17.5% of patients. Independent risk factors for restenosis, as determined by multivariate analysis, were stent diameter (OR 0.06, *p* = 0.05), arterial hypertension (OR 6.75, *p* = 0.05), and neutrophil count (OR 276.07, *p* < 0.001) [2]. In our study, the neutrophil count was also elevated due to the bacterial infection being associated with COVID-19 [14,15]. Another Chinese study found a significant correlation between coronary artery restenosis and left ventricular ejection fraction, as well as the number of stents placed [4]. A similar study found a statistically significant relationship between coronary artery restenosis and the triglyceride and glucose index (OR: 3.49, *p* = 0.0006) [3].

Some authors have linked coronary lesions associated with stent restenosis to the calcification of the vessel wall, as well as to an increased fatty attenuation index of the pericoronary arteries, which is associated with perivascular inflammation [16]. The results of our study also highlight the significant contribution of inflammatory processes to the development of coronary artery restenosis.

We assessed the short-term consequences of previous COVID-19 infection in the study groups based on the Kaplan–Meier analysis of overall hospital survival. Differences in overall survival during the hospital stay were statistically significant between the groups with and without previous COVID-19 infection (9.53 vs. 9.82 days, respectively). When assessing the risk of a fatal outcome depending on the presence of COVID-19 infection at the hospital stage, a fourfold increase in the risk of an unfavorable outcome was found in the group with COVID-19. These results suggest a higher risk of death in patients with repeated myocardial revascularization after COVID-19 infection and a lower survival rate for this group of patients.

When assessing the survival of patients over the course of a year, the average survival time in the group with a history of COVID-19 was statistically significantly lower compared to the group without COVID-19 (χ^2^ = 11.611; *p* < 0.001). The risk function analysis for developing an adverse outcome over the year showed a twofold increase in the risk of death in the group of individuals who had COVID-19. When assessing the relationship between overall survival and the risk factors under study using Cox regression, the adjusted risk ratio for adverse outcomes in patients with a history of COVID-19 was 2.017; with increasing age, the risk increased by 1.028 times. The relationship between ejection fraction and the risk of an adverse outcome was negative.

In the group of individuals with restenosis who had undergone CVI, the average survival time was minimal—309.5 ± 9.4 days [95% CI 290.9; 328.01]; in the group without restenosis, but with a history of CVI, this indicator was 327.7 ± 10.1 days [95% CI 307.9; 347.4]; while in the groups of individuals without CVI, there were no statistically significant differences (340.9 ± 5.1 [95% CI 330.9; 350.9] and 339.6 ± 4.2 [95% CI 331.4; 347.9], respectively). These data indicate that it was the coronavirus infection that contributed to the decrease in survival rates in patients requiring repeated myocardial revascularization, regardless of the presence of coronary artery restenosis.

The results of our study suggest that patients with a previously revascularized myocardium have a higher risk of stent restenosis after coronavirus infection due to excessive neointimal hyperplasia, hypercoagulability, inflammatory responses, and endothelial dysfunction. Coronavirus infection can be a trigger for restenosis mechanisms even in the late period after the disease due to the persistent inflammatory process in the vascular wall and the vascular remodeling associated with the activation of fibrosis processes [7,8].

Comparing our patient survival data with the existing literature was challenging due to the lack of similar publications in the PubMed database. However, some studies described coronary artery restenosis unrelated to COVID-19, or they were conducted on smaller sample sizes with limitations. For instance, a prospective cohort study involving 10,004 patients who underwent routine control angiography 6–8 months after coronary stenting found that the presence of restenosis during follow-up angiography predicted 4-year mortality [17].

In the United States, a study conducted in 2020 based on the National STEMI Inpatient Database included 159,890 cases. Of these, 2210 (1.38%) had concomitant COVID-19. Mortality rates were significantly higher in patients with COVID-19 (17.8% vs. 9.1%, OR 1.96, *p* < 0.001), and these patients had fewer opportunities for percutaneous coronary intervention on the day of hospital admission (63.6% vs. 70.6%, *p* = 0.019), as well as lower rates of coronary artery bypass grafting (3.0% vs. 6.8%, *p* = 0.008). Complications such as cardiogenic shock, cardiac arrest, acute renal failure, and stroke occurred with the same frequency among patients with and without COVID-19. Patients with STEMI and concomitant COVID-19 had significantly higher in-hospital mortality rates (almost twice as high) [12]. These findings were consistent with another large study of 1150 patients with myocardial infarction, which showed that COVID-19 co-infection was associated with significantly worse angiographic, procedural, and clinical outcomes. Patients with COVID-19 had longer hospital stays and higher mortality rates. Adjusting for other potential risk factors did not change the results, indicating an independent effect of COVID-19 on the studied parameters [13].

A more recent study in 2024 involving 555,540 patients with myocardial infarction, of whom 5818 had concomitant COVID-19, demonstrated that patients with COVID-19 were more likely to undergo thrombolysis but less likely to receive coronary angiography. These patients also had more frequent complications from myocardial infarction, higher odds of mortality, and longer hospital stays. The authors concluded that COVID-19 predicted a worse prognosis for hospitalized patients with myocardial infarction.

## 5. Limitations

The main limitations of our study were the tracking of vital status and adverse cardiovascular events during the year using telephone interview data, which could be associated with the loss of some information.

The patients included in the study had an average age of over 60 years, which was associated with a large number of comorbid conditions. We only considered the most common comorbid diseases as risk factors for cardiovascular events.

Another limitation of our study was the difficulty in determining the severity of coronavirus infection retrospectively from medical records and during a telephone conversation.

Another limitation was the small sample size and the fact that our study was a retrospective observational study, which provided a lower level of evidence compared to randomized trials and carried a higher risk of bias. In the future, a prospective study should be conducted to evaluate the contribution of various factors to the development of coronary artery restenosis, as well as to identify the factors influencing the survival of patients with coronary artery restenosis.

## 6. Conclusions

The results of our study showed that previous COVID-19 and CRP were the factors associated with coronary artery restenosis. The average survival time in the group with a history of COVID-19 was statistically significantly lower than in individuals without COVID-19, both during the hospital stay and within one year after repeated revascularization. The adjusted hazard ratio (HR) for adverse cardiovascular events was increased for both the presence of COVID-19 and age. As left ventricular ejection fraction (LVEF) increased, the risks of adverse outcomes were statistically significantly reduced.

## Figures and Tables

**Table 1:** Comparative analysis of factors associated with coronary artery stent restenosis in study groups.

Risk Factors	Group with In-Stent Restenosis (N = 420)		Group Without In-Stent Restenosis (N = 511)		*p*	Reference
	N	%	N	%		
Diabetes mellitus	91	201.7	100	19.6	0.430 ^a^	
Arterial hypertension	414	98.6	497	97.3	0.170 ^a^	
History of COVID-19	161	38.4	106	20.8	0.001 ^a^	
Male gender	315	75	385	75.3	0.904 ^a^	
Middle age	64.2 (56.0–72.4)		64.3 (56.2–72.4)		0.813 ^b^	
LVEF	52.0 (45.75–57.0)		52.0 (43.0–56.0)		0.455 ^b^	
D-dimer (ng/mL)	452.0 (295–619)		437.0 (293–613)		0.58 ^b^	0.0–550.0
Troponin I (mcg/L)	0.1 (0.1–0.26)		0.1 (0.1–0.28)		0.831 ^b^	0.017–0.05
ALT (U/L)	25.0 (17.47–35.95)		25.6 (18.0–37.9)		0.43 ^b^	0.0–32.0
AST (U/L)	23.1 (17.38–33.51)		23.52 (17.36–36.3)		0.681 ^b^	5.0–34.0
Creatinine (µmol/L)	85.25 (72.0–102.0)		87.0 (72.0–102.1)		0.794 ^b^	71.0–115.0
C-reactive protein (mg/L)	10.7 (5.97–17.55)		9.06 (4.5–17.78)		0.003 ^b^	0.10–7.0
Triglycerides (mmol/L)	1.67 (1.17–2.38)		1.6 (1.12–2.36)		0.677 ^b^	0.34–1.70
LDL (mmol/L)	2.78 (2.19–3.45)		2.78 (2.17–3.49)		0.882 ^b^	0.10–3.0

^a^—significance was determined by the χ^2^ criterion; ^b^—Mann–Whitney test; ALT—alanine aminotransferase; AST—aspartate aminotransferase; LVEF—left ventricular ejection fraction; LDL—low-density lipoprotein.

**Table 2:** Regression analysis of factors associated with coronary artery stent restenosis.

Risk Factors	B	OR	95% Confidence Interval		*p*
			Lower Limit	Upper Limit	
Diabetes mellitus	0.128	1.137	0.826	1.564	0.431
Arterial hypertension	−0.665	0.514	0.196	1.351	0.177
History of COVID-19	0.866	2.378	2.778	3.191	<0.001
Age	−0.001	0.999	0.986	1.012	0.858
Male gender	0.018	1.019	0.755	1.373	0.904
LVEF	0.004	1.004	0.990	1.018	0.570
D-dimer	0.000	1.0	1.000	1.000	0.799
Troponin	0.000	1.02	0.998	1.012	0.983
ALT	0.002	1.002	0.998	1.006	0.304
AST	0.001	0.999	0.998	1.000	0.686
Creatinine	0.001	1.001	0.999	1.002	0.953
C-reactive protein	0.009	1.009	1.0003	1.015	0.002
Triglycerides	−0.002	0.998	0.926	1.076	0.969
LDL	0.025	1.025	0.897	1.172	0.716

ALT—alanine aminotransferase; AST—aspartate aminotransferase; B—regression coefficient; LVEF—left ventricular ejection fraction; LDL—low-density lipoprotein; OR—Odds Ratio.

**Table 3:** Multiple regression analysis of the association between risk factors for stent restenosis.

Risk Factors	B	OR	95% Confidence Interval for OR		*p*
			Lower Limit	Upper Limit	
COVID-19	0.948	2.29	1.711	3.078	<0.001
C-reactive protein	0.077	1.08	1.002	1.013	0.012

**Table 4:** Characteristics of risk factors for adverse cardiovascular events depending on COVID-19 history.

Factors	COVID-19 History +, N = 269	COVID-19 History −. N = 662	*p*
Restenosis, n (%)	161 (60.3)	258 (39.0)	<0.001 ^a^
Age	64.0 (59.0–70.0)	64.0 (57.0–72.0)	0.824 ^a^
Sex: male, n(%)	193 (72.3)	506 (76.4)	0.486 ^b^
Arterial hypertension	179 (96.8)	300 (98.7)	0.189 ^c^
Diabetes mellitus	41 (22.2)	59 (19.4)	0.464 ^b^
Systolic BP	130.0 (120–141.5)	130.0 (120.0–141.5)	0.683 ^a^
Diastolic BP	80.0 (70.0–90.0)	80.0 (80.0–90,0)	0.179 ^a^
Heart rate	77.0 (70.0–82.0)	78.0 (70.0–81.50)	0.451 ^a^
LV EF, %	51.0 (46.0–56.0)	52.0 (46.0–56.0)	0.837 ^a^
Leukocytes	8.2 (6.48–10.6)	8.04 (6.7–10.30)	0.755 ^a^
Lymphocytes	24.9 (18.85–33.15)	25.0 (20.05–31.60)	0.830 ^a^
Neutrophils	65.0 (57.7–74.3)	65.35 (58.0–71.67)	0.764 ^a^
NLR	2.72 (1.75–4.03)	2.58 (1.89–3.48)	0.604 ^a^
PLR	122.83 (89.78–168.59)	115.06 (86.57–149.34)	0.033 ^a^
Platelets	234.0 (200.0–272.0)	231.0 (193.0–272.25)	0.157 ^a^
Hemoglobin	140.0 (128.0–152.0)	143.0 (132.0–153.25)	0.093 ^a^
APTT	31.3 (26.7–34.55)	28.50 (25.14–33.38)	<0.001 ^a^
INR	1.0 (0.9–1.1)	1.0 (0.93–1.1)	0.714 ^a^
Fibrinogen	3.30 (2.73–4.16)	3.14 (2.56–3.80)	0.014 ^a^
D-dimer	490.0 (346.3–714.0)	472.0 (282.25–594.00)	<0.001 ^a^
Troponin	0.1 (0.1–2.76)	0.1 (0.1–0.15)	<0.001 ^a^
ALT	27.0 (17.85–36.8)	24.2 (17.77–37.00)	0.295 ^a^
AST	25.0 (18.4–39.0)	23.0 (17.05–34.0)	0.002 ^a^
CPK	198.0 (148.5–370.0)	183.2 (102.6–274.5)	0.001 ^a^
MB CPK	23.1 (17.1–47.9)	18.0 (14.3–27.73)	<0.001 ^a^
Glucose	6.2 (5.43–8.66)	6.0 (5.4–7.38)	0.012 ^a^
Urea	5.75 (4.74–7.20)	5.8 (4.80–7.30)	0.775 ^a^
Creatinine	82.0 (71.0–101.35)	87.0 (72.63–102.0)	0.092 ^a^
CRP	11.8 (4.85–21.2)	8.27 (4.1–15.6)	0.005 ^a^
LDL	2.88 (2.19–3.48)	2.71 (2.17–3.47)	0.542 ^a^
HDL	0.99 (0.88–1.23)	1.02 (0.9–1.24)	0.456 ^a^
Triglycerides	1.60 (1.11–2.30)	1.65 (1.15–2.40)	0.666 ^a^

^a^—Mann–Whitney U-test; ^b^—Pearson Chi-square; ^c^—Fisher’s exact test. BP—blood pressure; CRP—C-reactive protein; LV EF—left ventricular ejection fraction; CPK—creatine phosphokinase; MB CPK – MB creatine phosphokinase; HDL—high-density lipoproteins; LDL—low-density lipoproteins; ALT—alanine aminotransferase; AST—aspartate aminotransferase, APTT—activated partial thromboplastin time; INR—International Normalized Ratio; NLR—neutrophil-to-lymphocyte ratio; PLR—platelet-to-lymphocyte ratio.

**Table 5:** Risk of developing adverse cardiovascular events depending on risk factors.

Risk Factors	Unadjusted Risk		Adjusted Risk	
	HR; 95% CI	*p*	HR; 95% CI	*p*
COVID-19	1.948; 1.319–2.879	<0.001	2.017; 1.364–2.981	<0.001
LV EF	0.972; 0.954–0.991	0.004	0.972; 0.953–0.991	0.004
Age	1.028; 1.008–1.049	0.005	1.028; 1.008–1.048	0.006

LV EF—left ventricular ejection fraction.

## Data Availability

The data are not publicly available due to confidentiality agreements and privacy concerns but can be accessed upon reasonable request to ensure proper use and adherence to ethical guidelines.

## References

[1] Megaly M., Alani F., Cheng C.I., Ragina N. (2021). Risk Factors for the Development of Carotid Artery In-Stent Restenosis: Multivariable Analysis. Cardiovasc. Revasc. Med..

[2] Liu D., Xue Z., Qi J., Yin L., Duan B., Wu L., Yang K., Gao B., Cao Q., Mi J. (2024). Risk factors for instent restenosis of sirolimus-coated stents in coronary intervention for patients with unstable angina. Sci. Rep..

[3] Liang S., Wang C., Zhang J., Liu Z., Bai Y., Chen Z., Huang H., He Y. (2023). Triglyceride-glucose index and coronary artery disease: A systematic review and meta-analysis of risk, severity, and prognosis. Cardiovasc. Diabetol..

[4] Li M., Hou J., Gu X., Weng R., Zhong Z., Liu S. (2022). Incidence and risk factors of in-stent restenosis after percutaneous coronary intervention in patients from southern China. Eur. J. Med. Res..

[5] Cortese B., Testa G., Rivero F., Erriquez A., Alfonso F. (2023). Long-Term Outcome of Drug-Coated Balloon vs Drug-Eluting Stent for Small Coronary Vessels: PICCOLETO-II 3-Year Follow-Up. JACC Cardiovasc. Interv..

[6] Sheng X., Yang G., Zhang Q., Zhou Y., Pu J. (2024). Impact of risk factors on intervened and non-intervened coronary lesions. Am. J. Cardiovasc. Dis..

[7] Asakura H., Ogawa H. (2021). COVID-19-associated coagulopathy and disseminated intravascular coagulation. Int. J. Hematol..

[8] Valencia I., Lumpuy-Castillo J., Magalhaes G., Sánchez-Ferrer C.F., Lorenzo Ó., Peiró C. (2024). Mechanisms of endothelial activation, hypercoagulation and thrombosis in COVID-19: A link with diabetes mellitus. Cardiovasc. Diabetol..

[9] Azova M., Timizheva K., Ait Aissa A., Blagonravov M., Gigani O., Aghajanyan A., Tskhovrebova L. (2021). Gene Polymorphisms of the Renin-Angiotensin-Aldosterone System as Risk Factors for the Development of In-Stent Restenosis in Patients with Stable Coronary Artery Disease. Biomolecules.

[10] Pivina L., Batenova G., Omarov N., Ygiyeva D., Messova A., Alibayeva G., Jamedinova U., Kurumbayev R., Pivin M. (2025). Peculiarities of in-Stent Thrombosis and Restenosis in Coronary Arteries Post-COVID-19: A Systematic Review of Clinical Cases and Case Series. Open Access Emerg. Med..

[11] Nanavaty D., Sinha R., Kaul D., Sanghvi A., Kumar V., Vachhani B., Singh S., Devarakonda P., Reddy S., Verghese D. (2024). Impact of COVID-19 on Acute Myocardial Infarction: A National Inpatient Sample Analysis. Curr. Probl. Cardiol..

[12] Goel A., Malik A.H., Bandyopadhyay D., Isath A., Gupta R., Hajra A., Shrivastav R., Virani S.S., Fonarow G.C., Lavie C.J. (2023). Impact of COVID-19 on Outcomes of Patients Hospitalized With STEMI: A Nationwide Propensity-matched Analysis. Curr. Probl. Cardiol..

[13] Mohsenizadeh S.A., Alidoosti M., Jalali A., Tofighi S., Salarifar M., Poorhosseini H., Jenab Y., Ahmadian T. (2022). Comparison of Angiographic and Clinical Outcomes After Primary Percutaneous Coronary Intervention for ST-elevation Myocardial Infarction Between Patients with and Without Concomitant COVID-19 Infection. Crit. Pathw. Cardiol..

[14] Pivina L., Batenova G., Ygiyeva D., Orekhov A., Pivin M., Dyussupov A. (2024). Assessment of the Predictive Ability of the Neutrophil-to-Lymphocyte Ratio in Patients with In-Stent Restenosis after COVID-19. Diagnostics.

[15] Gu N., Chen P., Wang X., Shen C., Deng Y., Chen J., Ma Y., Ma S., Hu X., Zhao R. (2024). Association Between the Neutrophil-to-Lymphocyte Ratio and in-Stent Neoatherosclerosis and Plaque Vulnerability: An Optical Coherence Tomography Study. J. Cardiovasc. Pharmacol..

[16] Adolf R., Krinke I., Datz J., Cassese S., Kastrati A., Joner M., Schunkert H., Wall W., Hadamitzky M., Engel L.C. (2025). Specific calcium deposition on pre-procedural CCTA at the time of percutaneous coronary intervention predicts in-stent restenosis in symptomatic patients. J. Cardiovasc. Comput. Tomogr..

[17] Lee O.-H., Hong S.-J., Ahn C.-M., Kim J.-S., Kim B.-K., Ko Y.-G., Choi D., Jang Y., Hong M.-K. (2019). The incidence of noncardiac surgery in patients treated with drug-eluting stents according to age. J. Invasive Cardiol..

